# Increasing control improves further control, but it does not enhance memory for the targets in a face–word Stroop task

**DOI:** 10.3758/s13421-020-01028-2

**Published:** 2020-03-06

**Authors:** Luis Jiménez, Cástor Méndez, Oscar Agra, Javier Ortiz-Tudela

**Affiliations:** 1 Universidad de Santiago de Compostela, Santiago de Compostela, Spain; 2 Goethe Universität, Frankfurt, Germany

**Keywords:** Cognitive control, Recognition memory, Conflict-driven memory enhancements, ISPCE

## Abstract

Recent research on the dynamics between attentional and memory processes have outlined the idea that applying control in a conflicting situation directly leads to enhanced episodic memory of the processed information. However, in spite of a small subset of studies supporting this claim, the majority of the evidence in the field seems to support the opposite pattern. In this study, we used a face–word Stroop task to enforce different control modes either from trial to trial or in an item-specific manner. Both manipulations of congruency proved to be effective in making participants’ responses to conflicting stimuli more efficient over time by applying a trial-specific control mode. However, these manipulations had no impact on memory performance on a surprise recognition memory test. To our knowledge, this is the first attempt at measuring the memory consequences of the application of specific control modes at the trial level. The results reported here call for caution and possibly reconceptualization of the relationship between cognitive control and memory.

Pursuing unusual goals (e.g., throwing a new sequence of punches when boxing or including new moves in your tango sequence) is more demanding than performing comparatively more habitual goals (e.g., sticking to your old moves in both scenarios) because to reach infrequent goals, performers have to take every step required to accomplish such goals and also prevent the potential intrusion coming from more habitual actions performed in those contexts. The processes recruited to overcome the conflict between alternative action courses are collectively referred to as “cognitive control,” and they have been explored systematically by means of interference lab tasks such as Stroop (MacLeod, 1992; Stroop, 1935), flanker (Eriksen & Eriksen, 1974), or Simon (Simon & Berbaum, 1990) tasks. For instance, in a Stroop task, if a participant is told to respond to a word denoting a color by referring to the color in which it is printed, its semantic content leads to an interference that is measured as the difference in reaction times between the conditions in which both features are congruent or incongruent with each other.

One important result from the literature on cognitive control is that the efficiency of control processes is not invariant, but is rather subject to systematic changes. Thus, the effect of congruency decreases immediately after responding to an incongruent trial (i.e., the congruency sequence effect, or CSE; Gratton, Coles, & Donchin, 1992), or after responding to a large proportion of incongruent trials over a given block (i.e., list-wide proportion congruency effect, or LWPCE; Logan & Zbrodoff, 1979), a specific context (context-specific proportion congruency effect, or CSPCE; Crump, Gong, & Milliken, 2006), or even for a specific item (item-specific proportion congruency effect, or ISPCE; Jacoby, Lindsay, & Hessels, 2003). Thus, it appears that the efficiency of cognitive control becomes finely attuned to the previous experience, and it improves precisely in those conditions in which it becomes challenged.

## Learning and the dynamics of cognitive control

One of the most prominent attempts to account for the control dynamics outlined above came from the conflict monitoring theory (CMT) proposed by Botvinick, Braver, Barch, Carter, and Cohen (2001). The CMT suggests that conflict signals generated on an incongruent trial trigger a temporal up-regulation of cognitive control that improves its focus on the target immediately after having encountered that conflicting trial. Adaptation to conflict can explain both CSE and LWPCE, assuming that the increase of control produced immediately after a conflict trial does not decline completely after a single trial, but tends to produce gradual and cumulative effects, modulating control over extended periods of training. However, the model has more problems dealing with control effects that appeared linked to specific contexts or specific types of trial. Temporal modulations such as those proposed by the CMT are not well suited to account for changes associated to specific features, which instead call for the acquisition of enduring associations between those features and the specific parameters of control that are requested under these circumstances (Blais, Robidoux, Risko, & Besner, 2007; Verguts & Notebaert, 2008).

One prediction that follows from the link between associative learning and cognitive control is that the increased control triggered by any experience of conflict should result not only in more efficient responses but also in an enhanced encoding of the relevant episodes. Specifically, if the original CMT claims that the adaptation process takes place across successive trials (Botvinick et al., 2001), such improvements in control could be expected to lead to an enhanced encoding of the episodes that come right after an incongruent trial, rather than of the event that generated the conflict. However, other interpretations have suggested that a full readjustment of control could take place within a single trial, thus supporting the prediction of an enhanced encoding of the conflicting episodes (Scherbaum, Fischer, Dshemuchadse, & Goschke, 2011). Other authors have claimed that the experience of conflict acts as a trigger for arousal responses, which improve the efficiency of associative learning and hence promote adaptation as a consequence of learning, rather than the other way around (Verguts & Notebaert, 2008). In any of these cases, regardless of whether associative learning plays an antecedent or consequent role in its dynamic relation with cognitive control, all these accounts predict a close relationship between control and memory.

## Control as memory

A few other theoretical accounts have attempted to explain this learning-adaptation dynamic by adopting a full-fledged episodic standpoint, thus conceiving the observed modulations of control as the effects of priming, derived from the reinstatement of the features of a previous trial, or of the repetition of the whole cognitive set that recurs after having experienced the same settings on an immediately previous trial. Thus, rather than assuming that conflict leads to control and thus to increased learning (Botvinick et al., 2001), or that conflict leads to learning, and hence to improved control (Verguts & Notebaert, 2008, 2009), the episodic accounts claim that all cognitive processes are primed by the reinstatement of the contexts in which they were implemented, and thus that fluctuations in performance reflect different instantiations of that rule. This episodic view was originally proposed by Mayr, Awh, and Laurey (2003) to account for the CSE as a result of repetition priming, and by Hommel, Proctor, and Vu (2004) to understand this phenomenon in terms of previous experience with particular feature bindings. A more global instantiation of the same idea was put forward more recently by Egner (2014), assuming that the control adjustments triggered in a particular context become incorporated into the episodic event files, thus binding the internal cognitive state and the attentional settings applied to that context together with the features of that episode; this binding makes it easier to apply the same settings in exactly the same ways when that particular stimulus configuration recurs. In any case, the question remains open with respect to whether such generalized binding processes can support exclusively an improvement in response to a close replication of the same task or whether it could also improve memory for the identity of the target.

## Conflict enhanced memory

The hypothesis of conflict-enhanced memory has been recently examined through several studies using different paradigms (Krebs, Boehler, De Belder, & Egner, 2015; Ortiz-Tudela, Milliken, Botta, LaPointe, & Lupiañez, 2016; Ortiz-Tudela, Milliken, Jiménez, & Lupiáñez, 2018; Rosner, D’Angelo, MacLellan, & Milliken, 2015a). For instance, Krebs et al. (2015) used a face–word Stroop task, in which participants were asked to respond to the gender of a given set of faces that were overlaid with a distracting word (i.e., “MAN” or “WOMAN”). These words rendered Stroop-like congruency effects, as the gender of the face could match or mismatch the meaning of the word; accordingly, the authors measured faster responses when the word accurately indicated the gender of the target face. More important for the current purposes, the authors also reported that when participants were later asked to perform a recognition memory test on the target faces, they produced a larger proportion of high-confident recognition responses to those faces that were paired with an incongruent distracter.

Rosner and colleagues reported a similar result (Davis, Rosner, D’Angelo, MacLellan, & Milliken, 2019; Rosner et al., 2015a; Rosner, Davis, & Milliken, 2015b) using a naming task, in which participants were presented with pairs of spatially interleaved words written in two different colors and were told to read aloud the word written in one of the colors. Participants were faster when both words were identical, but they were more able to recognize those target words that had been presented with an incongruent distracter.

At variance with these previous results, Ortiz-Tudela et al. (2018) tested up to seven variations of a spatial cueing paradigm in which participants were told either to read aloud or to categorize a long series of words preceded by a visual cue that generated a spatial expectation about the target location. Even though these experiments succeeded in generating and breaking spatial expectations, as judged by the congruency effects obtained in participants’ reaction times (RTs), the authors found no evidence consistent with the hypothesis that a mismatch of such spatial expectations could be enough to trigger any enhancement in memory.

## The present study

The main goal of this study was to further investigate the hypothesis that conflict enhances memory, going back to the original paradigm devised by Krebs et al. (2015), in an attempt to reproduce and extend the evidence gathered in that study. Because it is not clear whether the memory enhancement triggered by an upsurge of control should take place within a single trial (Scherbaum et al., 2011) or would be better expressed on the trial that immediately follows a conflicting trial, as originally proposed by the CMT (Botvinick et al., 2001), in Experiment 1, we conceptually replicated the original experiment, but assessed the effects of responding to a conflict trial (*n*) both on the recognition of the face presented on that trial (*n*) and on the face presented on the successive trial (*n* + 1), as compared with recognition of the face that immediately preceded the conflict trial(*n* − 1). To foreshadow the results, this experiment produced no evidence consistent with the hypothesis that conflict produced a generalized enhancement of memory, neither for the conflicting trial, nor for the trial that immediately followed that conflict, even though it suggested that memory may be selectively enhanced for those conflicting trials that come right after another conflict trial.

In Experiment 2, we modified the procedure in an attempt to strengthen any boost in encoding directly triggered by cognitive conflict. We reasoned that if enhanced encoding was indeed triggered by conflict, but in a very mild way, maybe a single presentation of a given face in incongruent conditions was not enough to produce a significant modulation of memory; we therefore aimed at increasing any potential effects by repeating the presentation of certain faces under conditions of high or low proportion of congruency. As described above, the ISPCE has been documented in several procedures (Bugg, Jacoby, & Chanani, 2011; Jacoby et al., 2003), indicating that participants can learn to associate particular control settings to specific stimuli when they are consistently presented in conditions of high versus low conflict. Applying this reasoning to the face–word Stroop task, we expected that a repeated exposure of particular faces in conditions of high versus low conflict could lead to (1) an adaptive modulation of control, as measured by the ISPCE, and (2) larger differences in memory performance between congruent and incongruent faces when those were presented in conditions of high versus low proportion of congruency.

## Experiment 1

The extent to which conflict-driven memory enhancements are restricted in time to the boost in encoding of the conflicting information or whether they could also affect information presented following the conflict is still unsolved. Accordingly, one prominent theory that explains CSE states that the detection of conflict between coactive representations triggers enhanced processing not only of the current event but also of subsequent stimuli (Gratton et al., 1992). In this experiment, we intended to test whether the up-regulation of cognitive control observed for *n*-lagged trials produces recognition memory benefits for the items in said trials. We used a face–word Stroop paradigm in which we measured congruency effects in recognition memory both for trial *n* and trial *n* + 1.

### Method

#### Participants

The original effect found by Krebs e al. (2015) was observed on a sample of 20 participants. An a priori power analysis based on the result of their matched-samples *t* test (*t* = 2.29) indicated that the size of the difference between the recognition of congruent and incongruent faces amounted to a Cohen’s *d* of .51, an effect size that the original sample of 20 participants would be able to catch with a power (1 − β) of only.71. To increase that power to the recommendable criterion of .80, we needed a sample of 28 participants. However, in order to increase that power to a target level of .90, we aimed at recruiting valid data from a total of 36 participants. We recruited 37 students from the Universidad de Santiago de Compostela to take part in the study. They signed informed consents and took part in the experiment in exchange for course credit. The study was part of a larger project that was approved by the local Ethical Committee of the University of Santiago de Compostela. One participant was removed from the sample because he or she did not understand the face gender task and simply watched the faces without responding to this task.

#### Stimuli

**Fig. 1:**
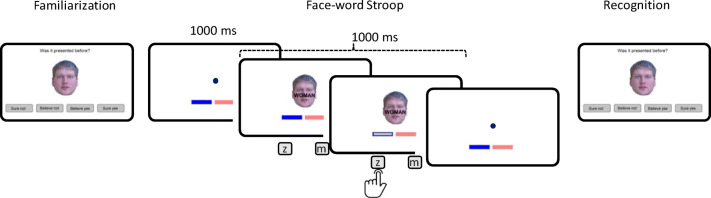
Schematic representation of Experiment 1 showing, from left to right, a trial from the familiarization task, the temporal arrangement of a trial from the face–word Stroop task, and a trial from the recognition task

The face stimuli were selected from the same database used by Krebs et al. (2015), the Glasgow Unfamiliar Face Database (Burton, White, & McNeill, 2010), that includes 304 stimuli corresponding to male and female faces, cropped to preserve exclusively the contour of the heads. From the overall sample, we performed an initial selection to exclude those that appeared especially distinctive, and selected a subset of 180 faces (90 male and 90 female) to be included in the study. In the familiarization and memory test phases, each face was presented alone, over a white background, with dimensions of approximately 5 × 7 cm (note that these dimensions varied slightly among different pictures, because their size is not completely uniform). In the face–word Stroop task, each face was overlaid with a congruent or an incongruent word (the Spanish words for *MAN* or *WOMAN*) written in black, Arial bold 24-point capitalized font (3.4 × 0.8 cm), located approximately over the nose area (see Fig. 1).

#### Procedure

To conceptually replicate the procedure of Krebs et al. (2015), participants were first presented with a familiarization task, followed by the face–word Stroop task, and a surprise memory test, which was administered after a distracter task in which participants were asked to perform an unrelated task for a period of approximately 15 min.^1^

##### Familiarization

In the familiarization task, participants had the first opportunity to view the faces that were going to appear later in the Stroop task. This was included in the experiment by Krebs et al. (2015) under the argument that responding to completely novel stimuli can reduce the interference caused by the irrelevant words, and to avoid floor effects in the memory test. Participants were asked to pay attention to the faces, and to indicate whether each face had been seen previously or not. In the present version of the task, participants used the computer mouse to click on four possible buttons represented as pictures at the bottom of the screen, that contained the legends “sure not,” “believe not,” “believe yes,” and “sure yes” to represent their response to the question of whether that particular face had been presented earlier or not. For each participant, the program randomly chose 72 out of the 90 pictures of male faces, and another 72 out of the 90 pictures of female faces, for a total of 144 faces, which were presented twice at random. Thus, an already-presented face could appear at every moment in the task, and participants were free to inspect the faces for as long as they wished before deciding on a response. The following trial appeared immediately after having responded to the previous trial, and the task continued up to the end of the 288 trials.

##### Face–word Stroop task

After the familiarization task, the full set of 144 familiarized trials were presented once again in the context of a face–word Stroop task. Each of these Stroop trials was preceded by a fixation point presented for 1000 ms, centered on the position in which the irrelevant word would appear overlaid on the face. Both face and word appeared later for another period of 1000 ms, and participants were told to indicate the gender of the face regardless of the meaning of the word, using the keys “Z” and “M” from a standard QWERTY keyboard. The specific mapping between gender and responses was counterbalanced across participants, and the particular mapping used for each participant was reminded to them by using two horizontal color bars (blue and pink) presented at the bottom of the screen at the relative locations corresponding respectively to male and female categories. Upon pressing a response key, the inner part of the bar corresponding to the chosen response turned grey, to provide an immediate feedback of the performed response. If an error was committed, or if no response was emitted before the end of the 1000 ms exposure time, a warning error sound was emitted. Critically, the word superimposed on each face could be either congruent with the face gender (i.e., the word WOMAN on a female face) or incongruent with it (the word MAN over a female face), and the overall proportion of congruency was 50%.

##### Recognition memory test

**Fig. 2:**
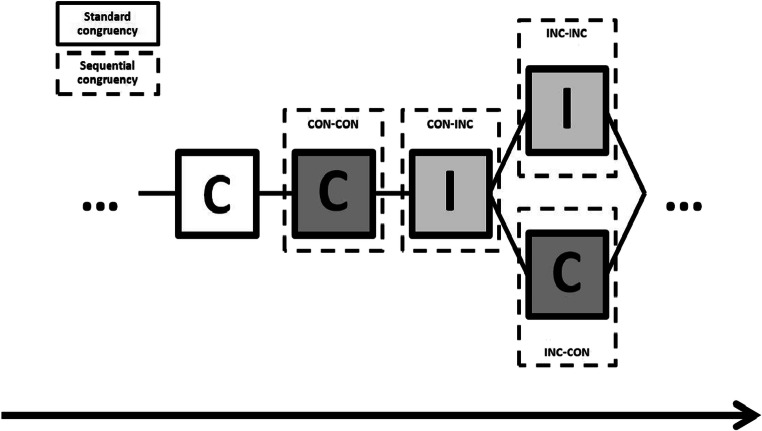
Depiction of the trial type transitions in Experiment 1. For sequential analyses, trials are labeled as a function of congruency transitions in the Stroop task. Capital C and capital I inside the squares refers to congruent and incongruent trials in the Stroop task, respectively. Both types of trials can either be followed by a congruent trial (i.e., top label CON-CON and INC-CON) or by an incongruent trial (i.e., top label CON-INC and INC-INC). Trials with a white background were not included in the analyses (see the text for a more detailed description)

After a delay of approximately 15 min, which was filled with an unrelated serial-reaction-time task, participants were presented with a surprise recognition memory test. On each of these trials, participants saw a face and were asked to judge whether it had been presented before or not, using the same categories employed during the familiarization task (i.e., “sure not,” “believe not,” “believe yes,” “sure yes”). The faces selected for the recognition task included 36 completely new faces (from here on, *NEW*) and four different types of faces already seeing in the face–word Stroop task. These old faces were automatically selected by the program to include all the incongruent faces that occurred after a series of at least two congruent trials (from here on, CON-INC), the congruent faces that immediately preceded each of these selected incongruent trials (and that therefore occurred after another congruent trial, CON-CON), and those faces that followed the referred incongruent trials, which were further subdivided as incongruent postincongruent (from here on, INC-INC) and congruent postincongruent (from here on, INC-CON; see Fig. 2 for visual depiction of the trial coding).

The number of trials contained in each category depended on the particular random distribution of trials generated for each participant, but it amounted to between 19 and 25 CON-INC and CON-CON trials, and the same number of postincongruent trials, which were evenly divided between INC-INC and INC-CON trials. This procedure allowed us to test recognition memory from a group of faces presented on closely neighboring trials, but differing specifically on the level of conflict experienced on that trial and on the preceding trial.

### Results

#### Familiarization phase

The familiarization phase was analyzed only to confirm that participants were performing the orienting task properly and to assess if the amount of time devoted to processing each face depended on whether it was new or repeated. Recognition responses of “sure not,” “believe not,” “believe yes,” and “sure yes” were coded as −2, −1, 1 and 2, respectively. Participants produced an average negative score of −0.91 for the first presentation of the faces, and a positive score of .62 for their second presentation. An ANOVA conducted on these scores showed that participants’ responses accurately discriminated between new and repeated faces, *F*(1, 35) = 402.08, *p* < .001, η_p_^2^ = .92. If recognition responses were simply taken at their qualitative value, either as “yes” or “no,” the average proportion of correct responses amounted to .76 for the new faces and .66 for the repeated faces. Both scores were significantly larger than those expected by chance, *t*(35) = 12.11, *p* < .001, for new faces, and *t*(35) = 7.24, *p* < .001 for repeated faces. Responses were also faster for repeated than for new faces (1675 vs. 1860 ms), *F*(1, 35) = 15.91, *p* < .001, η_p_^2^ = .31. Taken together, these results indicated that participants were aptly performing the familiarization task.

#### Face–word Stroop task

Participants’ performance in this task was generally fast (537 ms) and accurate (.91 of correct responses). Proportion of correct responses and response times (RTs) were submitted to separate analyses of variance (ANOVAs), with congruency of the current trial (congruent vs. incongruent) and congruency of the previous trial (previous congruent vs. previous incongruent) as within-participants factors. For the analyses of RTs, only correct trials were included.

A Stroop effect was found both on RT and accuracy measures, as participants responded faster (517 vs. 557 ms) and more accurately (.95 vs. .87) to congruent than to incongruent trials, *F*(1, 35) = 45.57, *p* < .001, η_p_^2^ = .57, and *F*(1, 35) = 42.86, *p* = .001, η_p_^2^ = .55, respectively, for RT an proportion of hits. The effect of previous congruency was also significant for RT, showing faster responses after a congruent trial (527 vs. 547 ms), *F*(1, 35) = 18.39, *p* < .001, η_p_^2^ = .34, but not for the measure of accuracy (.912 vs. .907), *F* < 1. Even though the numerical pattern suggested that congruency effects were larger after a congruent trial than after an incongruent trial (13 ms and 1.9 points in accuracy), the Congruency × Previous Congruency interaction was not significant in any of the analyses, *F*(1, 35) = 1.65, *p* = .21, η_p_^2^ = .05, for RT, *F* < 1 for accuracy.

#### Recognition memory test

**Fig. 3:**
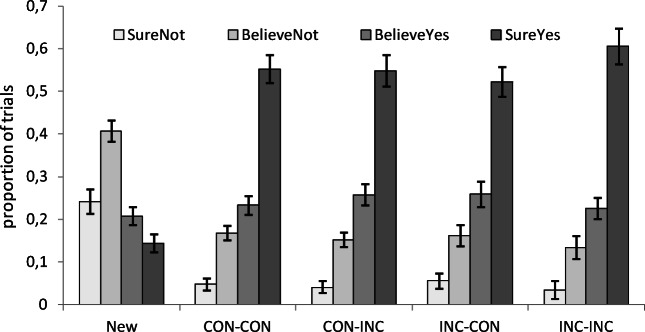
Recognition results, showing the proportion of “sure not,” “believe not,” “believe yes,” and “sure yes” judgments in response to faces not previously presented (New), as compared with old faces appearing in a congruent trial after another congruent trial (CON-CON), in an incongruent trial after a congruent trial (CON-INC), in a congruent trial after an incongruent trial (INC-CON), and in an incongruent trial after another incongruent trial (INC-INC). Error bars represent the standard error of the means

As shown in Fig. 3, the results indicate that participants’ recognition responses discriminated clearly between new and old faces, but that much smaller differences were found among the patterns observed in response to all the remaining types of trials. In keeping with the analyses conducted by Krebs et al. (2015), we focused on participants’ high-confidence recognition responses, as we expected to obtain an improved proportion of such responses specifically for faces presented under incongruent conditions. A preliminary analysis comparing the average proportion of high-confidence recognition responses for the full set of old faces as compared with those provided in response to new faces clearly showed that participants were able to discriminate between these two types of faces, *F*(1, 35) = 218.33, *p* < .001, η_p_^2^ = .86 (.55 vs. .14).

Once adequate overall memory performance was ensured, we turned to our main analyses of interest: memory performance as a function of current and previous trial congruency at encoding. In contrast with Krebs et al.’s (2015) result, an overall comparison of the proportion of high-confidence responses across all congruent and incongruent trials showed no significant difference between them, *F*(1, 35) = 1.58, *p* = .22, η_p_^2^ = .04. Participants recognized incongruent faces (.563) slightly more frequently than incongruent faces (.540), but a Bayesian analysis suggested that the evidence in favor of a difference between them was merely anecdotal, BF_10_ = .456.

For the analysis of Previous Congruency × Current Congruency interaction, we analyzed the proportion of high-confident recognition responses to old faces presented either in a congruent or in an incongruent Stroop trial, as a function of the congruency of the preceding Stroop task. An ANOVA with congruency and previous congruency as repeated measures showed no effect of previous congruency (*F* < 1), but it showed both a significant Congruency effect (.537 vs. .577), *F*(1, 35) = 4.23, *p* = .047, η_p_^2^ = .11, and a significant Congruency × Previous Congruency interaction, *F*(1, 35) = 5.58, *p* = .024, η_p_^2^ =.14. This interaction showed a higher rate of high-confident recognition responses to faces presented under incongruent conditions selectively when they followed another incongruent trial (.523 vs. .606), *F*(1, 35) = 7.37, *p* = .011, η_p_^2^ = .17, but not when they occurred after a congruent one (.551 vs. .548), *F* < 1. Bayesian paired *t* tests confirmed that there was moderate evidence for the effect of congruency on recognition responses after an incongruent trial, BF_10_ = 4.05, but not after a congruent trial, BF_10_ = 0.18.

### Discussion

The results of Experiment 1 showed clear congruency effects during the face–word Stroop task, but, unexpectedly, they failed to show a reliable CSE. Interestingly, the recognition task showed no evidence consistent with the hypothesis that conflict produced a generalized enhancement of memory, for either the conflicting trial or for those trials that come immediately after conflict. As noted in the introduction, this conflict-enhanced memory effect has been previously observed in similar paradigms, but the main aim of Experiment 1 was to assess whether the up-regulation of control provoked by an incongruent trial could lead to a better remembering of the face presented during the conflicting trial, or of the ones presented in the following trial. Contrary to both hypotheses, we found significantly higher recognition scores only for those incongruent trials that immediately followed another incongruent trial, thus suggesting that conflict over two successive trials might be required to trigger an effective increase in recognition.

In hindsight, the failure to replicate the recognition benefit found by Krebs et al.’s (2015) can be taken as less of a surprise if we consider two empirical and conceptual arguments. First, on purely empirical grounds, one should notice that the behavioral effect reported by Krebs et al. was relatively small, and it was statistically significant only in the context of a multiple series of planned *t* tests that compared the proportion of high-confident recognition responses given to a set congruent, neutral, and incongruent trials, without any correction for multiple comparisons. Second, on more conceptual grounds, one must take into account that recognition memory performance in this procedure can be driven not only by encoding those faces during the face–word Stroop task but also by processing of the same faces during the previous familiarization task. Note that Krebs et al.’s (2015) design and ours include a new set of faces as lures for the recognition memory phase, and thus it is impossible to disentangle effects of the two encoding phases. Moreover, if a conflict-driven enhancement is indeed taking place but is weak in nature, it is therefore possible that a single presentation of the faces in congruent or incongruent conditions is not enough to override the effects of the familiarization task. In Experiment 2, we introduced two main changes intended to increase the impact of conflict on the measures of memory: first, we made the conflict manipulation stronger, by presenting the faces repeatedly under congruent or incongruent conditions, and, second, we made the memory task dependent exclusively on the experiences gathered within the conflict task.

## Experiment 2

Experiment 2 aimed at further exploring the puzzling result of Experiment 1 by testing whether repeated exposure of faces under congruent or incongruent conditions could produce differences in remembering by virtue of triggering an additive encoding boost. To achieve this main goal, we used the same core paradigm of Experiment 1, with the following changes: First, during the Stroop task, we repeatedly presented the faces under conditions of either high or low conflict, to reinforce any possible effect of conflict-enhanced memory produced by a single presentation. In order to strengthen any congruency effect, we also moved the presentation of the distracter word earlier in time (Appelbaum, 2009). Second, only a subset of familiarized faces was passed along to the Stroop task, and the remaining ones were used as lures in the memory test; we therefore changed from a pure recognition memory task to a source memory task (Konopka & Benjamin, 2009). On doing so, we achieved the dual goal of avoiding any functional ceiling effect that could have been reached if participants were presented with a simple recognition task after having experienced multiple exposures to a reduced group of faces, and making recognition performance depend exclusively on the experience accumulated in the conditions of high versus low conflict.

Finally, to ensure that our manipulation effectively affected the amount of control exerted on each trial, we included a cognitive control manipulation that has been shown to modulate the degree of control at the item level. Instead of repeating some faces exclusively under incongruent conditions and others only under congruent conditions, we manipulated the proportion of congruency in three probabilistic levels for different items and assessed whether the items that appeared most frequently under incongruent conditions produced (1) smaller congruency effects in the Stroop trials and (2) higher levels of recognition in the memory test, as compared with those presented most frequently under congruent conditions.

### Method

#### Participants

Twenty-seven students from the Universidad de Santiago de Compostela signed informed consents and took part in the experiment in exchange for course credit.

#### Stimuli

The face stimuli were selected out of the same sample of 180 faces from the Glasgow Unfamiliar Face Database (Burton et al., 2010). In this experiment, the dimensions of the face pictures and the overlaid words were reduced with respect to those used in Experiment 1 (3 × 4 cm approximately for the faces, and 2.6 × 0.5 cm for the irrelevant words), and the pictures representing the response buttons appeared above instead of below the faces.

#### Procedure

The structure of Experiment 2 mimicked that of Experiment 1, with the following changes. The familiarization phase included two repetitions of only 50 faces, for a total of 100 trials. The faces were chosen at random for each participant, with the constraint that 25 of them corresponded to male faces and the remaining 25 corresponded to female faces. From these 50 familiarized faces, 15 male and 15 female faces were selected to be used in the face–word Stroop task, whereas the remaining 20 faces were reserved as lures for the recognition task. The face–word Stroop task was composed of two blocks of 120 trials, each presenting four repetitions of the 30 selected faces. These faces were further subdivided in three groups, each containing five male and five female faces, which were assigned respectively to conditions of high proportion of congruency (75/25), balanced congruency (50/50), and low proportion of congruency (25/75). Thus, each face from the face–word Stroop task was presented four times per block for a total of eight presentations. Ten of these faces were presented in a balanced proportion (i.e., they occurred four times in either congruent and incongruent trials), another 10 faces were presented mostly in congruent trials (six congruent vs. two incongruent presentations), and a final group of 10 faces were presented in mostly incongruent trials (two congruent and six incongruent trials). Therefore, although the overall proportion of congruency over the whole experiment was kept constant, a third of the trials belonged to “mostly incongruent” items, another third to “mostly congruent” items, and the final third to a balanced, control group.

On each Stroop trial, the trial started with an 800-ms fixation point. This was replaced by the distracter word (i.e., the Spanish word for *MAN* or *WOMAN*), which remained on the screen for 200 ms, and it was then replaced by the combination of the target face plus the same superimposed word. This remained on the screen for another 1000 ms, regardless of participants’ response. As in Experiment 1, participants responded using the keys “Z” and “M,” with the mapping between gender and responses counterbalanced across participants. Participants received visual feedback of their response, but the faces remained on the screen until the end of the trial. They also received a warning sound whenever they committed an error.

After the Stroop task, participants performed another distracter task,^2^ and then they were presented with the surprise recognition task. In this task, participants were presented with the full series of 50 faces presented during the familiarization task and were instructed to recognize which of them had been also presented during the gender discrimination task, using the same four categories of responses to represent their relative confidence in their response. Note that in this version of the memory test, a “new” correct response would imply a face not presented in the Stroop task, although all of the faces where included in the familiarization task.

### Results

#### Familiarization task

As in Experiment 1, we confirmed that participants were performing the orienting task and assessed the amount of time devoted to processing each face depending on whether it was presented for the first or for the second time. The average score for the first presentations of the faces was of −1.29, while the score for the second presentation was 1.02, *F*(1, 26) = 576.88, *p* < .001, η_p_^2^ = .96. The average proportion of qualitatively correct (i.e., yes or no) recognition responses amounted to .84 and .73, respectively, for new and repeated faces, both of them being significantly larger than those expected by chance, *t*(26) = 17.38, *p* < .001, for new faces, and *t*(26) = 12.53, *p* < .001, for repeated faces. Responses were also faster for repeated than for new faces (2729 vs. 2406 ms), *F*(1, 26) = 4.91, *p* = .04, η_p_^2^ = .15. Thus, participants were able to perform the familiarization task according to what could be expected.

#### Face–word Stroop task

Participants’ performance in this task was fast (544 ms) and accurate (.94 of correct responses). Accuracy (proportion of correct responses) and RTs were submitted to separate ANOVAs with congruency of the current trial (congruent vs. incongruent), congruency of the previous trial (previous congruent vs. previous incongruent), and item type (mostly congruent, balanced, and mostly incongruent) as within-participants factors. For the analyses of RT, only correct trials were included.

**Fig. 4:**
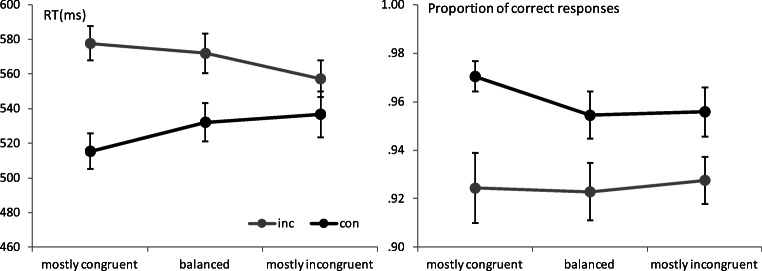
Reaction times (left panel) and proportion of correct responses (right panel) obtained in response to faces presented under congruent (con) and incongruent (inc) conditions, represented separately for those faces that appeared with the same frequency in each of these conditions (balanced), and for those that appear more often under either congruent or incongruent conditions. Error bars represent the standard errors of the means

A Stroop effect was found both on RT and accuracy measures, as participants responded faster (528 vs. 569 ms) and more accurately (.96 vs. .925) to congruent than to incongruent trials, *F*(1, 26) = 120.53, *p* < .001, η_p_^2^ = .82, and *F* (1, 26) = 14.11, *p* = .001, η_p_^2^ = .35, respectively. The effect of previous congruency was also significant for RT (543 vs. 554 ms), *F*(1, 26) = 8.49, *p* = .007, η_p_^2^ = .25, but not for the measure of accuracy (.942 vs. .943), *F* < 1. The main effect of type of item was not significant for either RT or accuracy (*F*s < 1). Interestingly, there were two interactions that reached significant levels in the analysis of RT: The Congruency × Previous Congruency interaction, *F*(1, 26) = 18. 93, *p* < .001, η_p_^2^ = .42, revealed that congruency effects were larger after a congruent trial than after an incongruent trial (55 vs. 27 ms). Although this effect was not significant in the analysis of accuracy, *F*(1, 26) = 3.18, *p* = .09, η_p_^2^ = .11, the pattern also indicated that the numerical difference in accuracy was larger after a congruent than after an incongruent trial (4.6 vs. 2.5 points), thus confirming that the effect observed in RTs was not the result of a speed–accuracy trade-off. Most importantly, the Congruency × Item Type interaction was also significant, *F*(2, 52) = 9.89, *p* = .001, η_p_^2^ = .28, indicating that the congruency effect observed for balanced items (39 ms, *p* < .001) grew larger for mostly congruent items (62 ms, *p* < .001) and decreased for mostly incongruent items (21 ms, *p* = .004). Bonferroni-corrected comparisons indicated that these differences were largely due to faster responding to the incongruent trials in the mostly incongruent condition, as compared with the balanced and mostly congruent conditions (15 ms, *p =* .02, and 20 ms, *p* = .006, respectively), as well as to an improved responding to congruent trials under mostly congruent conditions, when compared with balanced (17 ms, *p* = .029), but not to mostly incongruent conditions (21 ms, *p* = .10). In sum, the results were consistent with the claim that the item type manipulation did effectively modulate the amount of control exerted in response to each face. Although this interaction was nonsignificant in the analysis of accuracy (*F* < 1), the qualitative pattern pointed to the same trend to show larger effects of congruency for those items that were presented more often under congruent conditions (4.6) than for those presented in either balanced or mostly incongruent conditions (3.2 and 2.8 points, respectively; see Fig. 4).

#### Recognition

**Fig. 5:**
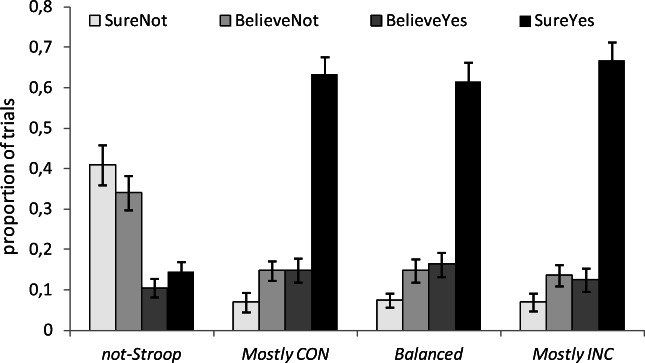
Source memory results, showing the proportion of “sure not,” “believe not,” “believe yes,” and “sure yes” judgments in response to faces not presented in the Stroop task (not-Stroop), as compared with faces presented mostly on congruent trials (Mostly CON), equally on congruent and incongruent trials (Balanced), and mostly on incongruent trials (Mostly INC). Error bars represent the standard error of the means

As shown in Fig. 5, the results indicated that participants’ recognition responses discriminated clearly between those faces that were presented repeatedly in the Stroop task and those that had been seen only in the familiarization phase, but that smaller differences were found among the patterns observed in response to all remaining types of trials. An analysis comparing the average proportion of high-confidence recognition responses to all faces presented in the Stroop task as compared with those exclusively seen in the familiarization phase clearly showed that participants were able to perform this source memory task, *F*(1, 26) = 182.32, *p* < .001, η_p_^2^ = .88 (.64 vs. .14). However, a comparison of the proportion of high-confidence recognition responses provided to those faces that appeared in the Stroop task, in terms of the relative frequency with which they were presented under congruent or incongruent conditions, indicated that participants’ responses did not discriminate among these three conditions, *F*(2, 52) = 1.05, *p* = .36, η_p_^2^ = .04. Participants recognized those faces presented more often on incongruent trials slightly better than those presented most frequently on congruent trials (.667 vs. .633), but this difference was far from significant, *t*(26) = 0.84, *p* = .41. A Bayesian *t* test provided a Bayes factor of B_10_ = .28.

In order to gather a potentially more sensitive measure of recognition to compare the three types of old faces, we adopted the scoring procedure described for the familiarization task, assigning scores of −2, −1, 1, and 2, respectively, to the responses “sure new,” “believe new,” “believe old,” and “sure old,” and we conducted an analysis of the effect of the amount of conflict on the average recognition scores obtained for each of the three types of old faces. The average scores corresponding respectively to mostly congruent, balanced, and mostly incongruent trials amounted respectively to 1.13, 1.10, and 1.18. A one-way ANOVA conducted on these scores did not produce a significant effect of type of face, *F*(2, 52) = 0.31, *p* = .73, η_p_^2^ = .01, and a Bayesian repeated-measures analysis produced a BF_01_ = 7.29, suggesting that the results were 7 times more compatible with the null hypothesis than with the existence of a genuine difference among the levels of recognition of these three types of faces. In other words, the results reinforced the conclusion that the conflict manipulation during encoding did not produce a differential recognition memory effect.

### Discussion

The results of this experiment showed a clear ISPCE, suggesting that participants are sensitive to the congruency context in which they have encountered a relatively long series of previously seen faces. The results showed that, even though participants responded faster to congruent than to incongruent trials, this difference was modulated by the previous experience with those faces, so that they responded faster to each specific face in the congruence condition in which it had been most frequently encountered. Reduction in RT specific for each face in its most frequent condition necessarily entails some form of learning (or of long-term priming) that should be responsible for the facilitation obtained when participants have to respond again to a particular combination of a face and an amount of conflict. However, the fact that participants improved their way of dealing with the particular congruency conditions associated with a face does not appear to rely on a better encoding of the face identity; in other words, the ISPCE effect was obtained in conditions that did not produce any differential effect in recognition performance. Indeed, the results of this experiment clearly indicate that increasing the amount of control needed to process a given face by exposing it repeatedly in conditions of high conflict does not result in larger recognition scores than those obtained by exposing participants to those faces in conditions of lower conflict. Plainly, then, the results are inconsistent with the claim that processing stimuli in conditions of high conflict should lead to later enhanced memory performance.

## General discussion

The conflict-driven memory enhancement hypothesis predicts that under circumstances of increased control, strong encoding of the processed information takes place. By having to actively boost processing of the relevant features of the stimuli (or by suppressing the irrelevant ones), attended information gets stored in such a way that it would be more easily accessed later on. The exact mechanism by which this “strong encoding” would act is still unknown. In this study we made use a face–word Stroop task to further explore the nature of this process. In two experiments, we manipulated the congruency between the gender of a face and a superimposed word. This manipulation being done at different levels allowed us not only to measure standard congruency effects block-wise but also to explore other manifestations of cognitive control, such as CSE and ISPCE.

In spite of obtaining reliable immediate effects (i.e., RT and accuracy) of face–word congruency, those effects did not generally transfer to memory performance. First of all, in the two experiments presented here, congruency measured during the current trial did not translate to better recognition scores for incongruent items. Second, in Experiment 1, congruency of the previous trial, which typically affects performance during the Stroop task, did not generally improve memory for all trials that followed an incongruent trial, even though we found evidence suggesting that responding consecutively to two incongruent trials might have produced an effect in the expected direction. Lastly, and perhaps more interestingly, manipulations of congruency at the item level, which undoubtedly imply learning about specific items, still did not affect our participants’ ability to recognize old items in any differential way. The absence of statistically significant differences when using null hypothesis significance testing, together with the use of Bayesian statistics to assess the likelihood of a null result in the presence of a true effect, greatly supports the claim that conflict at encoding does not directly lead to a better encoding of the target information (see Muhmenthaler & Meier, 2019; Ortiz-Tudela et al., 2016; Ortiz-Tudela et al., 2018; Ptok, Thomson, Humphreys, & Watter, 2019, for similar findings).

Several accounts of cognitive control depict LWPCE and CSE as a reinstatement of a previous response set linked with specific stimulus features. It can be very appealing to equate this reinstatement to a “memory enhancement.” However, caution is needed when making such claims. First, the dynamic interactions between cognitive control and learning processes are certainly a central aspect of human cognition, and cognitive control can be differentially applied as a function of past experience (Blais et al., 2007; Crump et al., 2006; Gratton et al., 1992; Jacoby et al., 2003; Verguts & Notebaert, 2008). However, the mechanisms that give rise to the response on a memory test, such as recognition, could very well be independent of these dynamics. Second, the assumption that heightened attention to relevant information in conflicting situations leads to better encoding of the attended information is very likely to be missing a key point: During (and after) conflicting situations a myriad of cognitive processes need to take place in order to overcome the interference. Some of these processes are likely to be harmful for memory encoding (e.g., directing attention towards the response set rather than to the stimulus itself), whereas others can indeed be beneficial (e.g., longer processing time). This mixed combination of processes could be responsible for the seemingly inconsistent pattern of results found in the literature. Of particular relevance here are postresponse processes such as performance monitoring or stimulus reelaboration that could be less important on congruent situations in which fluency in processing is predominant.

The two experiments reported in the present manuscript plus a substantial portion of the reviewed evidence supports the absence of a benefit for memory when a conflict is presented at encoding (by either measuring no differences or by actually finding the opposite). However, this set of results seems to be at odds with another substantial group of studies that was indeed able to measure a conflict-driven boost in memory performance (Krebs et al., 2015; Rosner et al., 2015b). However, this contradiction might not be such when examined carefully. In addition to subtle differences between the conflict tasks and the memory tests used in each of these studies, which could be responsible for the mixed pattern of results, we should also point out that most of the increased sensitivity showed for incongruent trials in the paradigm used by Rosner and colleagues occurred for false-alarm rates and not for hit rates (Davis et al., 2019, but see Rosner et al., 2015a, Experiment 2). In other words, rather than showing increased recognition of those ensembles shown under incongruent conditions, their results mostly showed that participants tended to falsely judge as old many of the new displays that were presented under congruent conditions. Because this could be explained by the increased processing fluency afforded by congruent ensembles, it will be worth examining whether these differences also arise systematically in hit rates.

Finally, it is worth noting that, although our experimental design is close to that of Krebs et al. (2015), some minor differences between the two studies prevent ours to be an exact replication; however, testing an exact replication was far from our intention. Indeed, the use of fMRI in the study by Krebs et al. (2015), in which they measured higher recognition performance for incongruent trials, enforces a slow task pace in which the intertrial interval (ITI) ranged from 1 to 7 s. Because this timing would be strange and inefficient for a behavioral experiment, and the entire context of an fMRI experiment includes many more factors that are impossible to recreate in a behavioral set up, we decided to opt for a conceptual replication. By staying close to their design in terms of the type of stimuli and type of conflict used, but using much more standard timing and experimental conditions for behavioral paradigms, we believe that the conclusions of our study can be more generalizable and thus more easily linked to other work with somewhat similar paradigms and goals.

It is, of course, possible that the differences between Krebs et al.’s (2015) results and ours (namely, the presence vs. absence of the conflict-driven memory effects) critically rely on the slow and variable pace of their study, but this would also point to the real breadth and scope of their results. More plausibly, perhaps, although still overtly speculative, one might adduce that the differences between Krebs et al.’s and our results might rely on the different procedures used to select the specific trials that were tested in the recognition task. Krebs et al. selected their recognition trials completely at chance, and therefore it was likely that their sample contained a comparable number of incongruent trials presented at encoding after another incongruent trial (i.e., INC-INC transition trials) than of trials in which the incongruent trial was presented after a congruent trial (i.e., CON-INC trials). In contrast, our selection procedure took as reference the incongruent trials that appeared after two congruent trials, and then selected their neighbors for comparison purposes. As a consequence, our incongruent test trials would include roughly a half of trials encoded in an INC-INC transition than trials encoded in the context of a CON-INC transition. If a memory improvement was driven by responding to several incongruent trials in a row, this effect could explain why Krebs et al. found an overall improvement for their incongruent trials, and why we failed to reproduce the same effect. Interestingly, in Experiment 1, memory for INC-INC trials was enhanced with respect to all other conditions. This was not a result that we expected, and therefore we must be cautious in offering a possible explanation for it. In principle, one might speculate that this pattern could point to the existence of a genuine effect of increased control in memory, but an effect that would be too small or inconsistent after a single incongruent trial to be reliably measured. As a consequence, one would need an uninterrupted series of control demanding trials to produce an up-regulation of cognitive control that would in turn change memory storage. This could explain why a test that assesses recognition from a random sample of faces could have found significant effects that our procedure was not able to replicate. A random selection of the faces presented over the Stroop task could have sampled a good number of faces that occurred in a context of two, three, or more previous incongruent trials; in contrast, our selection criteria tested only those faces that occurred on incongruent trials that came after two congruent trials in a row, and the trials that preceded and followed these referential trials. Perhaps the use of such strict criteria might have prevented cumulative effects from playing a role in our design. This so-far purely speculative hypothesis would need to be empirically pursued in future studies, perhaps manipulating the runs of congruent and incongruent trials, and measuring the impact of that manipulation in recognition performance. In any case, the fact that in Experiment 2 we did not find better memory for faces presented up to six times under incongruent conditions at least indicates that a single conflicting trial does not improve memory in a small and nonsignificant way that could be made significant by accumulating the impact of several encounters. In sum, the results of the present study are more consistent with the conclusion that one would need to accumulate a sufficient amount of conflict, by means of a series of incongruent trials, to produce an upsurge in memory storage.

Future research will probably need to take into account many other differences in experimental paradigms to fully explain this mixed pattern of results, and to clarify the mechanisms underlying these effects. Classic accounts of memory formation, such as the levels of processing framework (Craik & Lockhart, 1972) or the desirable difficulty hypothesis (Bjork & Bjork, 1992) could accommodate these conflict-driven boosts in memory performance. However, these theories lack a satisfying mechanistic explanation of the effect. Moreover, asymmetric effects without a clear intermediate/neutral condition that could be used as baseline can be equally interpreted as benefits or costs with very different implications for their underlying mechanisms.

## Conclusion

One could argue that the set of results presented here raises more questions than it provides definitive answers for (as it is often the case with null results). However, we firmly believe that what we have presented here is a direct test of a highly relevant and currently debated hypothesis. If the exertion of cognitive control necessarily triggers enhanced memory encoding of the processed information, its effect ought to be measurable following its most common manifestations. In the present study we have shown three different ways (i.e., current congruency, previous congruency, and item-specific proportion congruency) in which applying cognitive control improves performance in an immediate and time-restricted delayed way, but that do not render long-lasting effects in memory. Even in the conditions in which we can reliably measure that a specific enhanced control mode was learned and applied for specific items, this enhanced control was not directly translated to better encoding of identity of the item.

## Footnotes

1 In the original study, participants performed an fMRI task and other tasks unrelated to the study for about an hour. We presented them with a pilot study involving a serial-reaction-time task, in which participants were required to use four keys from the keyboard to respond to the location of a stimulus that moved over a series of four possible locations, following a probabilistic sequence. Participants performed six blocks of 96 trials with this task.

2 Distracter tasks in Experiment 2 consisted of a serial-reaction task. The task was distributed in two parts, one presented between the familiarization and the Stroop task, and another between the Stroop task and the recognition task. Specifically, participants were trained with a probabilistic serial-reaction-time task during the first delay interval, and were retrained with another block of the same task, and performed a generation task over the second delay interval. The intervals were variable depending on participants’ individual performance, but they took between 10 and 15 min on average.
